# Research trends and focus of prosthetic joint infections from 2013 to 2023: bibliometric and visualization studies

**DOI:** 10.3389/fmicb.2024.1507340

**Published:** 2024-12-20

**Authors:** Liwen Zhang, Fei Li, Diqian Zhao, Lei Duan, Wenzhe Bai, Bing Yan

**Affiliations:** ^1^The First Clinical Medical School, Shandong University of Traditional Chinese Medicine, Jinan, China; ^2^Department of Orthopedics, Affiliated Hospital of Shandong Traditional Chinese Medicine University, Jinan, China

**Keywords:** artificial joint infection, bibliometrics, biofilm, antibiotic resistance, infection diagnosis antimicrobial coating, infection control strategies

## Abstract

**Background:**

Postoperative infections in artificial joints provide considerable difficulties in the field of orthopedics, especially after joint replacement procedures. These infections rank among the most severe postoperative consequences, frequently leading to treatment ineffectiveness and reduced quality of life for surgery patients. Consequently, it is crucial to acquire knowledge about worldwide research trends in this area in order to educate clinical practices and improve therapeutic techniques. This work exploits bibliometric analysis to investigate the present state, developing patterns, and main areas of focus in research on artificial joint infection.

**Objective:**

To analyze the research trends, hotspots, and international collaborations on artificial joint infections worldwide from 2013 to 2023.

**Methods:**

Extractions of raw data were made from the WoSCC (Web of Science Core Collection) database. Detailed information collected includes the quantity of publications, authors, citations, publication year, h-index, references, country/region, journal, and keywords. Analysis of the data was conducted using VOSviewer version 1.6.10.0 and CiteSpace version 6.3.R1.

**Results:**

A total of 1,799 articles published between 2013 and 2023 were included in this analysis, showing a steady increase in publication with the United States leading at 553 articles. Infection rates and topics such as biofilm formation and antimicrobial resistance were highly cited, with Mayo Clinic contributing 65 articles as the most prolific institution.

**Conclusion:**

Research on biofilm infections, antibiotic resistance, and new biomarkers is a key focus, particularly on disrupting biofilms and enhancing diagnostics. There’s growing attention in biomarkers like *α*-defensins and exosomal miRNAs for PJI diagnosis, pointing to new clinical uses. Studies on antimicrobial-coated prosthetics and topical agents are also gaining importance in treatment strategies.

## 1 Introduction

As an effective treatment for end-stage joint diseases (e.g., osteoarthritis, rheumatoid arthritis, etc.), total joint arthroplasty (TJA) has greatly improved patients’ quality of life and motor function. However, despite the remarkable clinical outcomes of TJA, its postoperative complications, especially prosthetic joint infection (PJI), remain an important factor affecting the success rate of the procedure and patient prognosis (Borchardt and Tzizik, 2018). Prosthetic joint infection (PJI) is a devastating complication after prosthetic arthroplasty that can lead to postoperative joint pain, prolonged hospitalization, need for multiple surgeries, dysfunction, and even death. Although some progress has been made in recent years in terms of causative microorganisms, diagnostic criteria, preventive strategies, and therapeutic regimens for PJI, rapid and accurate diagnosis of PJI and reduction of the incidence of postoperative PJI are still hot and difficult issues in the field of artificial joint replacement (McNally et al., 2021). At present, the diagnosis of prosthetic joint infection (PJI) relies on serologic testing, joint fluid tests, bacteriologic cultures, and imaging tests. However, these diagnostic techniques face challenges in terms of precision and the interpretation of found data. Despite the availability of various diagnostic tools, there is currently no single test that can diagnose PJI with 100% accuracy, so we need to integrate results from multiple tests (Zhou et al., 2024).

The application of bibliometric analysis provides a methodological framework for quantitatively examining and assessing the dominant literature corpus in a given field (Mayr and Scharnhorst, 2014). The process of bibliometric analysis allows capturing key data including authors, keywords, journals, countries, institutions and references. As a result, this method of analysis is able to chart the trajectory of a field (Abramo et al., 2018). Bibliometric analysis, enhanced by modern computing, uses graphical and visual representations to strengthen literature reviews (Ma and Xi, 1992). Using CiteSpace and VOSviewer together leverages their strengths in producing knowledge graphs. CiteSpace applies set theory for data normalization, using specialized algorithms to create time-zone and timeline views, visualizing knowledge development over time. This approach highlights evolutionary patterns and emerging trends within a domain (Chen, 2006). VOSviewer uses a probabilistic approach to data normalization, offering visualizations for keywords, institutions, and co-authors. Its intuitive and visually appealing network, coverage, and density analyses are key features (Van Eck and Waltman, 2010).

## 2 Materials and methods

### 2.1 Data collection

Web of Science has been widely accepted by researchers as a high-quality database of digital literature resources and is considered as the most appropriate database for bibliometric analysis. In this study, Web of Science (Core Collection) was selected as the data source, and comprehensive and accurate retrieval of data was ensured by selecting SCI-EXPANDED and SSCI indexes. The defined search strategy was (TS = (“Prosthetic Joint Infections”) OR TS = (“Periprosthetic Joint Infections”) OR TS = (“Artificial Joint Infections”) OR TS = (“Joint Implant Infections”) OR TS = (“Postoperative Joint Infections”) OR TS = (“Infectious Arthritis”) OR TS = (“Prosthesis-Related Infections”) OR TS = (“Orthopedic Implant Infections”) OR TS = (“Orthopedic Implant Infections”) OR TS = (“Biofilm-Associated Joint Infections”)).

**Figure 1:**
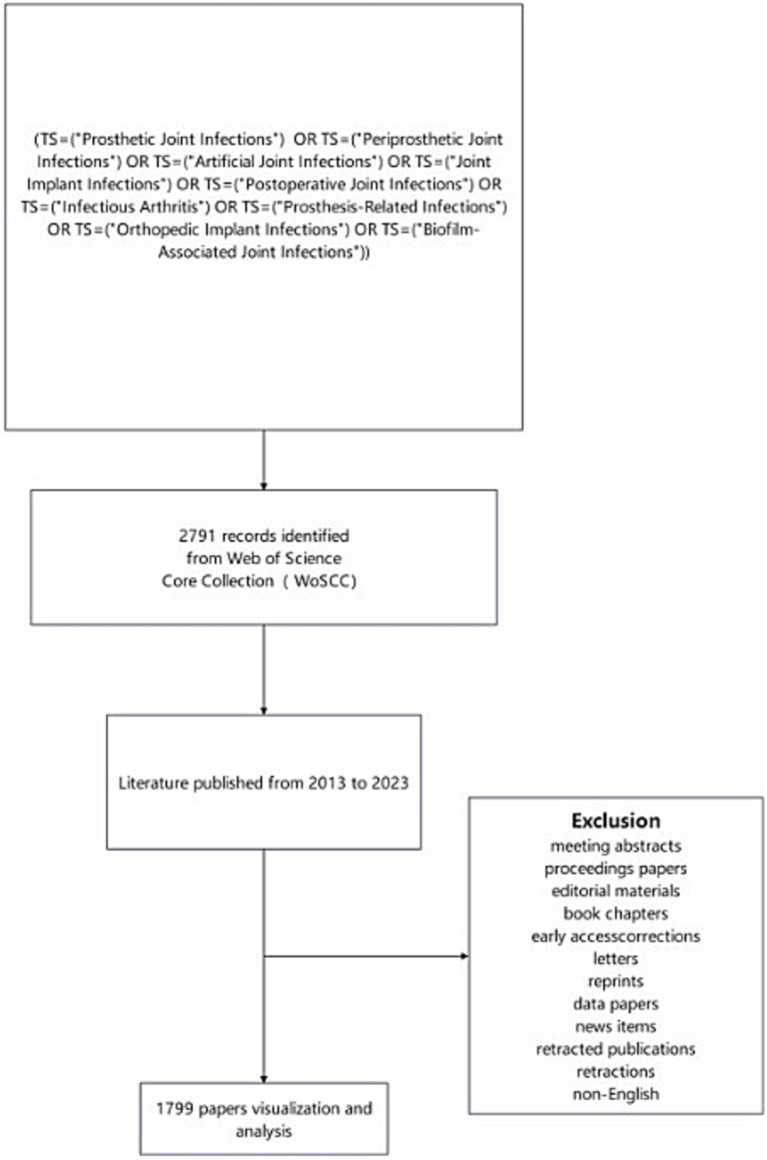
Flowchart of the research.

The time span was from 2013 to 2023 and the search deadline was July 14, 2024. Literature types were limited to articles and review articles, and the language restriction was English. A total of 1,799 journal articles were obtained after automatic de-duplication using CiteSpace 6.3.R1. The survey strategy used is shown in Figure 1.

### 2.2 Data analysis

All papers in the study were retrieved from the WoSCC database and comprehensively analyzed and visualized using VOSviewer and CiteSpace. CiteSpace integrates techniques from information visualization, bibliometrics, and data mining to identify trends and patterns in citation data (Synnestvedt et al., 2005). VOSviewer 1.6.10.0 was used to visualize the density distribution of authors, institutions, countries/regions, keyword clusters, co-cited references, authors, journals, and timelines.

### 2.3 Bibliometric analysis

Bibliometric indicators such as number of publications (Np) and number of citations (Nc) are crucial measures used to quantify the extent of the literature. The present study employed the metric of publications (Np) to evaluate productivity, and the metric of citations (Nc) to measure impact, as these two dimensions are fundamental in evaluating the extent of research. Co-citation is defined as the joint citation of a pair of items by a third independent item. Furthermore, the keyword co-occurrence statistic measures the frequency with which specific keywords are found together in the same academic publication (Merigó et al., 2018). The H-index is a metric that integrates productivity and citation impact by establishing a threshold that calculates the ratio between the number of publications (Np) and the number of citations (Nc) (Koseoglu et al., 2016). Therefore, when a researcher publishes H papers, each of which garners a minimum of H citations, they acquire an H-index of H. This is the minimum value for the H-index statistic (Hirsch, 2005). In particular, the H-index can be used to evaluate the academic achievements of an individual, as well as to reflect the intellectual productivity of a journal, association, country, or region (Molinari and Molinari, 2008). The impact factor (IF) is calculated based on the Journal Citation Reports (JCR) and is generally acknowledged as the main measure for evaluating the influence and excellence of scholarly publications (Zhou et al., 2022). The project involved the generation of bibliometric mapping using VOSviewer and CiteSpace, employing co-occurrence and co-citation analysis to provide a more thorough comprehension of the data.

## 3 Results

### 3.1 Overview of publications on PJI

Using a comprehensive search strategy, we identified 1,799 articles and reviews published between 2013 and 2023. The cumulative number of citations (NC) for these publications amounted to 31,304, with an average citation rate of 20.21 citations per document. The collective H-index of all identified publications was determined to be 76.

### 3.2 Annual trend of publication quantity

**Figure 2:**
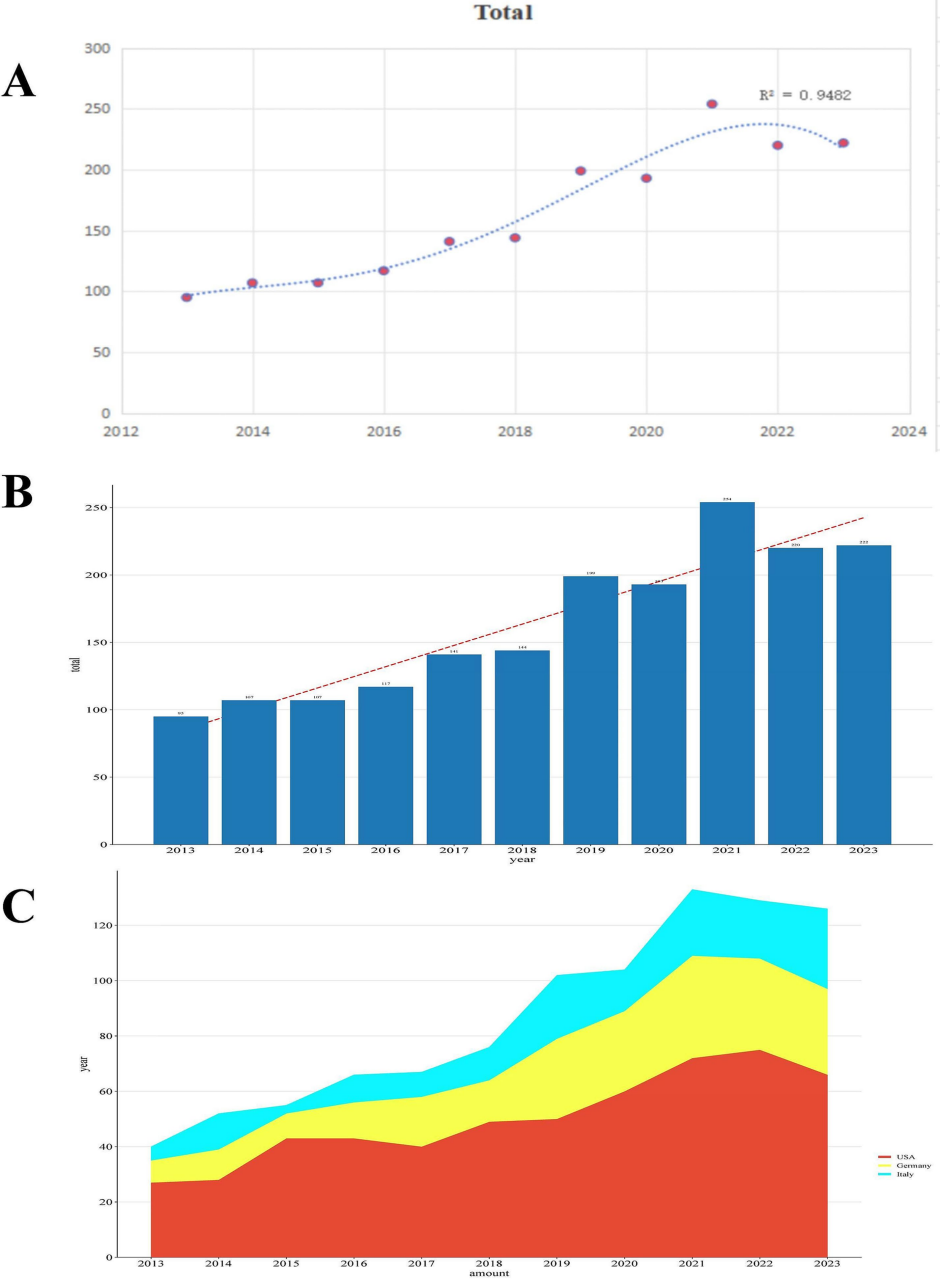
**(A)** Curve fitting of the total annual growth trend of publications. **(B)** Number of publications by year from 2013 to 2023. **(C)** Stacked area chart of the top three countries by publication volume from 2013 to 2023.

Figure 2 shows a strong correlation (r^2^ = 0.9482) between the number of publications (Np) and publication year. From 2013 to 2023, annual publications rose steadily from 95 to 222, peaking at 254 in 2021. The United States consistently led in Np, far surpassing other nations. Germany experienced steady growth until 2019, followed by a significant increase until 2021, and subsequently a downturn. Italy’s publication numbers fluctuated significantly, showing unpredictable growth. Research interest in prosthetic joint infections rose annually from 2013 to 2021, then declining. Notwithstanding recent oscillations, sustained expansion in this domain is anticipated, especially in prevention, diagnosis, and treatment.

### 3.3 Contributions of countries/regions

Table 1 presents the rankings of the top 10 nations and regions in artificial joint infection research. The United States leads with 553 publications, highlighting its dominance and influence in prosthetic joint infection studies. Although Germany has fewer publications, its 4,187 citations and an H-index of 37 demonstrate the strong impact of its research. Mainland China ranks fifth in publication volume, but its relatively low average citations suggest a need to improve research quality and influence. Spain, with a similar publication count to China, has a higher average citation rate, indicating better research quality. Switzerland stands out with the highest average citation count (36.09), despite a smaller number of papers, signaling strong recognition of its research impact.

**Table 1:** Top 10 productive countries/regions.

Rank	Country	NP	NC	H-index	Average citation per item
1	USA	533	12,408	54	23.54
2	GERMANY	233	4,187	37	18.99
3	ITALY	164	2,778	32	17.81
4	FRANCE	152	2,632	29	18.24
5	PEOPLES R CHINA	120	1,399	21	11.86
6	SPAIN	118	2,501	29	22.44
7	ENGLAND	107	3,179	30	30.39
8	NETHERLANDS	83	1,796	24	22.28
9	SWITZERLAND	76	2,689	28	36.09
10	CANADA	52	654	13	12.63

Overall, these statistics highlight distinctive scholarly contributions and impacts. While Germany, Italy, France, and Spain perform well in publication numbers, further efforts to boost research influence are needed. In contrast, England, the Netherlands, and Switzerland, despite fewer publications, exhibit high average citation rates, reflecting the quality and influence of their work. Mainland China and Canada should focus on improving both the quality and impact of their research alongside increasing publication output.

**Figure 3:**
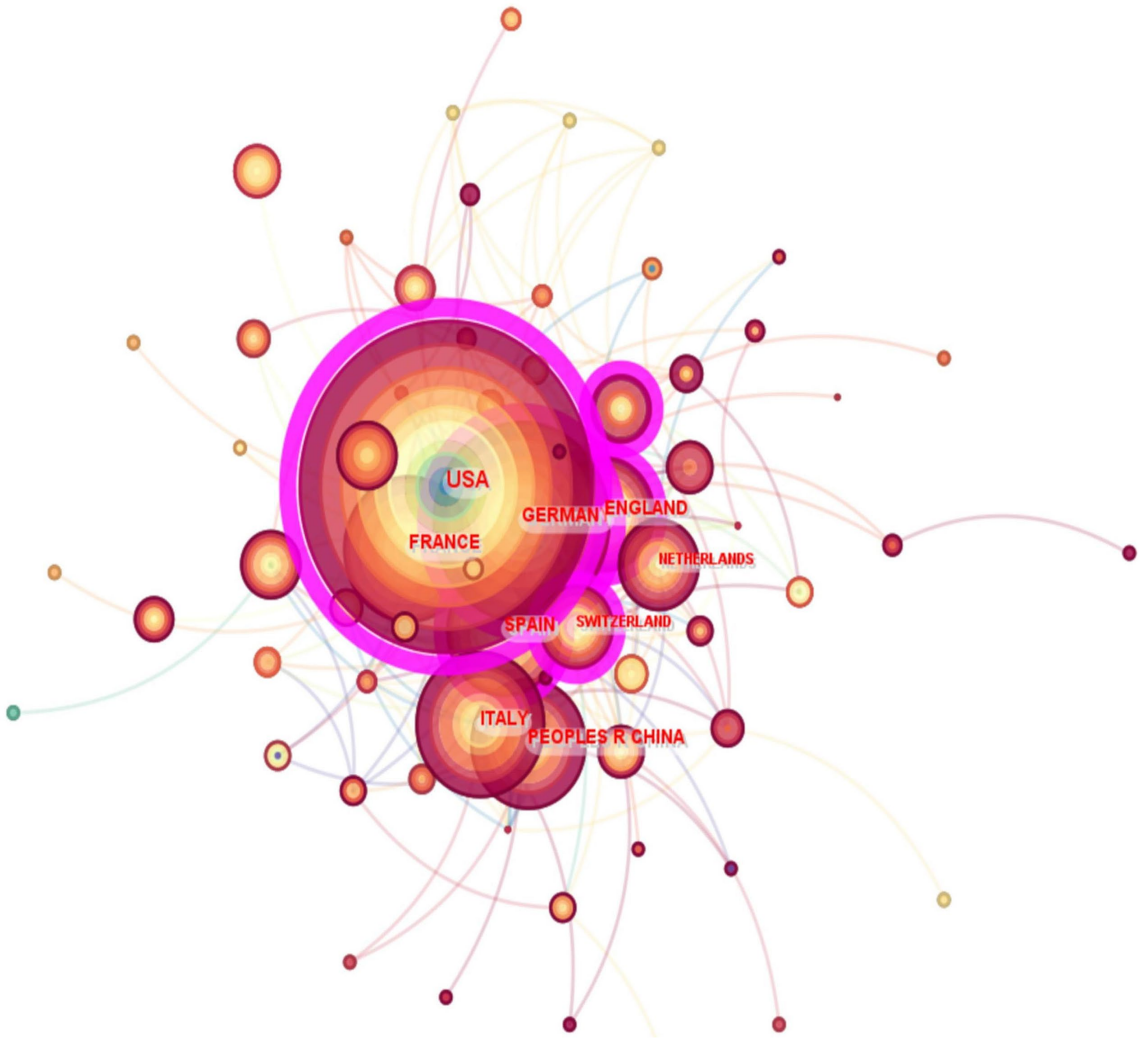
Mapping of countries of studies related to PJI.

The graphic map in Figure 3 illustrates the distribution of publications pertaining to artificial joint infections throughout several nations. Every node corresponds to a country, and the size of the node is directly proportionate to the corresponding number of outputs. Furthermore, the quantity of citations is directly proportional to the magnitude of the label assigned to each node. The borders between these circles delineate the level of collaboration among nations or institutions. The broader the boundaries, the more robust the cooperation. Node centrality in a knowledge graph is a metric that quantifies the importance of nodes and reveals the patterns of relationships among nodes. The purple circle delineates nodes that exhibit a significant level of centrality. In addition, the circle within the node denotes the quantity of product releases. Originating from a specific nation at a certain year, with distinct hues symbolizing several years. As depicted in Figure 3, a deeper hue of the circle corresponds to a lower year of publishing, while a lighter hue corresponds to a future publication. The analysis revealed two nodes with high centrality in terms of the number of recent publications on artificial joint infections: the United States (centrality = 0.50) and Germany (centrality = 0.31). Therefore, these nations are the primary partners in the science of artificial joint infections.

### 3.4 Analysis of affiliations

**Figure 4:**
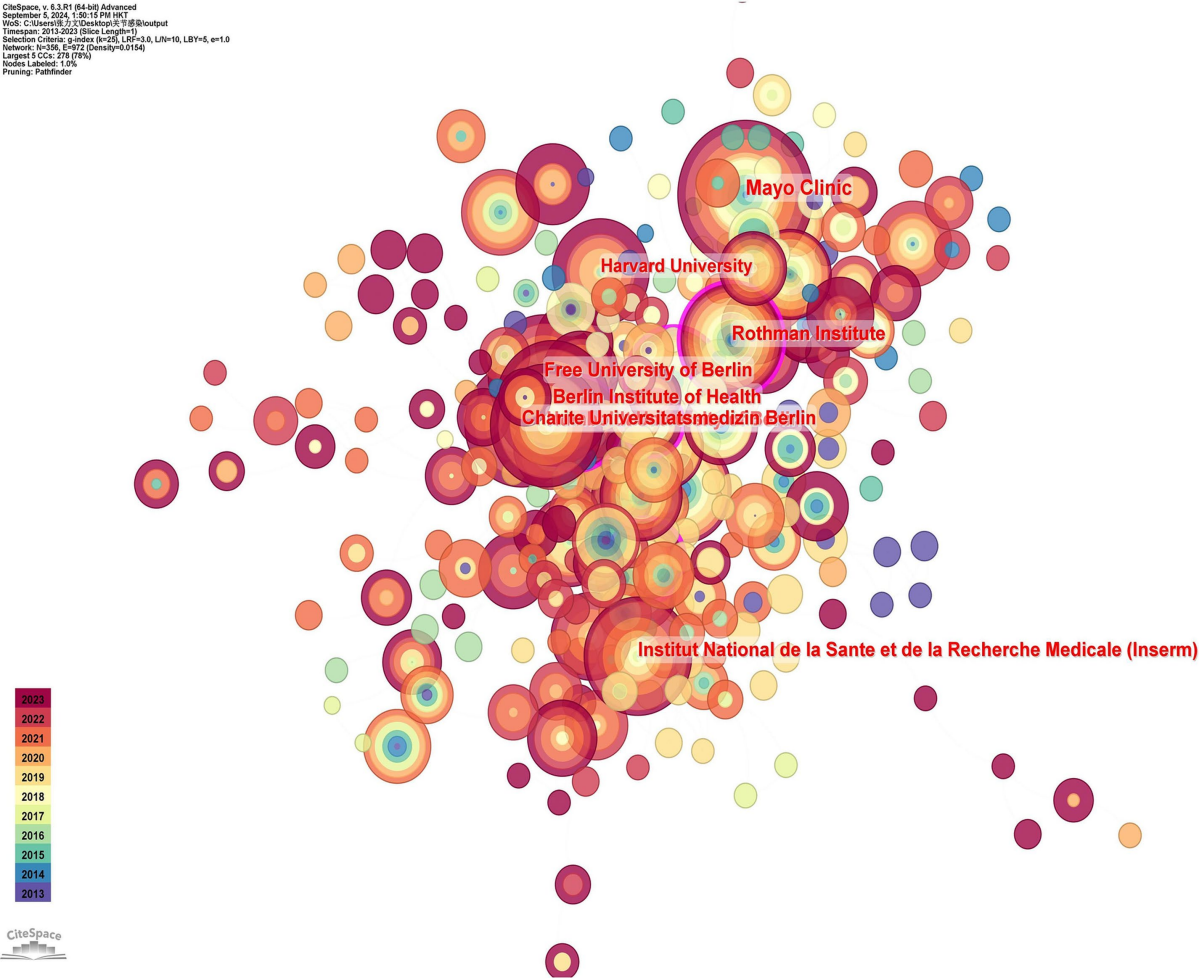
Mapping of countries of studies related to PJI.

Table 2 ranks the major institutions in the study of prosthetic joint infections according to the volume of publications. The Mayo Clinic (Mayo) led the way with 65 papers, followed closely by the Freie Universität Berlin. Although the University of Barcelona ranked relatively low in terms of the number of publications, it had the highest average number of citations (36.84), which can be attributed to the wide range of research topics at the University of Barcelona that dealt with several aspects of the epidemiology, microbiology, clinical manifestations, and diagnosis of infections of the prosthetic joint. The dominance of American institutions demonstrates the leadership of the United States in the academic field. Although INSEAD (Institute of National Superior of Administration and Management) leads in terms of publications, other indicators favor Rothman, which shows that a single metric cannot fully assess academic excellence. As shown in Figure 4, the color ranges from purple to red for the time lapse from 2013 to 2023. The Mayo Clinic Research Institute is the most productive institution, but its centrality is relatively low (centrality = 0.03). All of the top 10 publishing organizations have low centrality.

**Table 2:** Top 10 productive affiliations.

Rank	Affiliation	Country	NP	NC	H-index
1	MAYO CLINIC	USA	65	1,623	24
2	FREE UNIVERSITY OF BERLIN	GERMANY	55	1,380	23
3	HUMBOLDT UNIVERSITY OF BERLIN	GERMANY	54	1,333	23
4	CHARITE UNIVERSITATSMEDIZIN BERLIN	GERMANY	53	1,304	22
5	INSTITUT NATIONAL DE LA SANTE ET DE LA RECHERCHEMEDICALE INSERM	FRANCE	50	676	16
6	ROTHMAN INSTITUTE	USA	44	1,510	23
7	HARVARD UNIVERSITY	USA	43	720	16
8	JEFFERSON UNIVERSITY	USA	40	1,327	22
9	UNIVERSITY OF BARCELONA	SPAIN	38	1,349	19
10	UNIVERSITY OF MILAN	Italy	32	775	18

### 3.5 Performance of authors

The top 10 authors have collectively contributed 266 papers, which accounts for approximately 14.79% of the total submissions, as highlighted in Table 3. At the helm is Parvizi, Javad from Turkey, who has authored 36 papers and boasts the highest citation count, totaling 1,351. Senneville Eric is a close second with 34 papers and an impressive 892 citations. Notably, Soriano Alex, despite having a smaller number of papers, has made a significant impact with 1,243 citations, and stands out with the highest average citation count per paper at an outstanding 47.52. This underscores his considerable influence in the field.

**Table 3:** Top 10 authors with the most publications.

Rank	Author	Affiliation	Country	NP	NC	H-index	Average citation peritem
1	Parvizi, Javad	Acibadem University	TURKIYE	36	1,351	0 0	38.03
2	Senneville, Eric	Universite de Lille	FRANCE	34	892	18	26.76
3	Esteban, Jaime	Fundación Jiménez Díaz University Hospital	SPAIN	29	555	11	20.76
4	Trampuz, Andrej	Charite Universitatsmedizin Berlin	GERMANY	28	879	16	32.11
5	Soriano, Alex	Hospital Clinic Barcelona	SPAIN	27	1,243	7	47.52
6	Soderquist, Bo	Orebro University	SWEDEN	26	411	13	16.88
7	Mont, Michael a.	Sinai Hospital of Baltimore	USA	25	983	11	39.36
8	Randau, Thomas M.	University of Bonn	GERMANY	21	405	11	20.67
9	Drago, Lorenzo	IRCCS Multimedica	ITALY	20	511	13	26.95
10	erka, Carsten	Universitätsmedizin Berlin	GERMANY	20	436	10	22.5

### 3.6 Analysis of journals

In Table 4, the distribution of publications related to prosthetic joint infections across journals is detailed, highlighting the significant concentration of research in a small number of academic journals. The “JOURNAL OF ARTHROPLASTY” led the way with 179 papers and 3,401 citations, suggesting that it may be the preferred platform for the publication of such research. Despite not having the most publications, "CLINICAL ORTHOPAEDICS AND RELATED RESEARCH” stood out with an average of 53.52 citations per article, suggesting a significant impact despite the small number of articles. The impact factors of “JOURNAL OF BONE AND JOINT SURGERY AMERICAN VOLUME” (4.4) and “ANTIBIOTICS BASEL” (4.3) indicate that they are widely cited. Although some journals publish more articles, journals like “JOURNAL OF BONE AND JOINT SURGERY AMERICAN VOLUME” and “ANTIBIOTICS BASEL” demonstrate higher academic impact. This helps researchers to choose where to publish their work.

**Table 4:** Top 10 most active journals.

Rank	Journal	IF (2023)	NP	NC	H-index	Average citation per item
1	JOURNAL OF ARTHROPLASTY	3.4	179	3,401	32	19.92
2	ANTIBIOTICS BASEL	4.3	63	545	12	8.89
3	INTERNATIONAL ORTHOPAEDICS	2	52	975	19	19.37
4	JOURNAL OF ORTHOPAEDIC RESEARCH	2.1	34	1,090	13	32.32
5	ARCHIVES OF ORTHOPAEDIC AND TRAUMA SURGERY	2	33	266	8	8.3
6	CLINICAL ORTHOPAEDICS AND RELATED RESEARCH	4.2	33	1,761	22	53.52
7	JOURNAL OF ORTHOPAEDIC SURGERY AND RESEARCH	2.8	30	325	9	10.97
8	JOURNAL OF ANTIMICROBIAL CHEMOTHERAPY	3.9	27	720	14	26.96
9	JOURNAL OF BONE AND JOINT SURGERY AMERICAN VOLUME	4.4	27	709	14	26.48
10	PLOS ONE	2.9	27	984	15	36.56

### 3.7 Research hotspots, keywords analysis, research hotspots analysis

**Figure 5:**
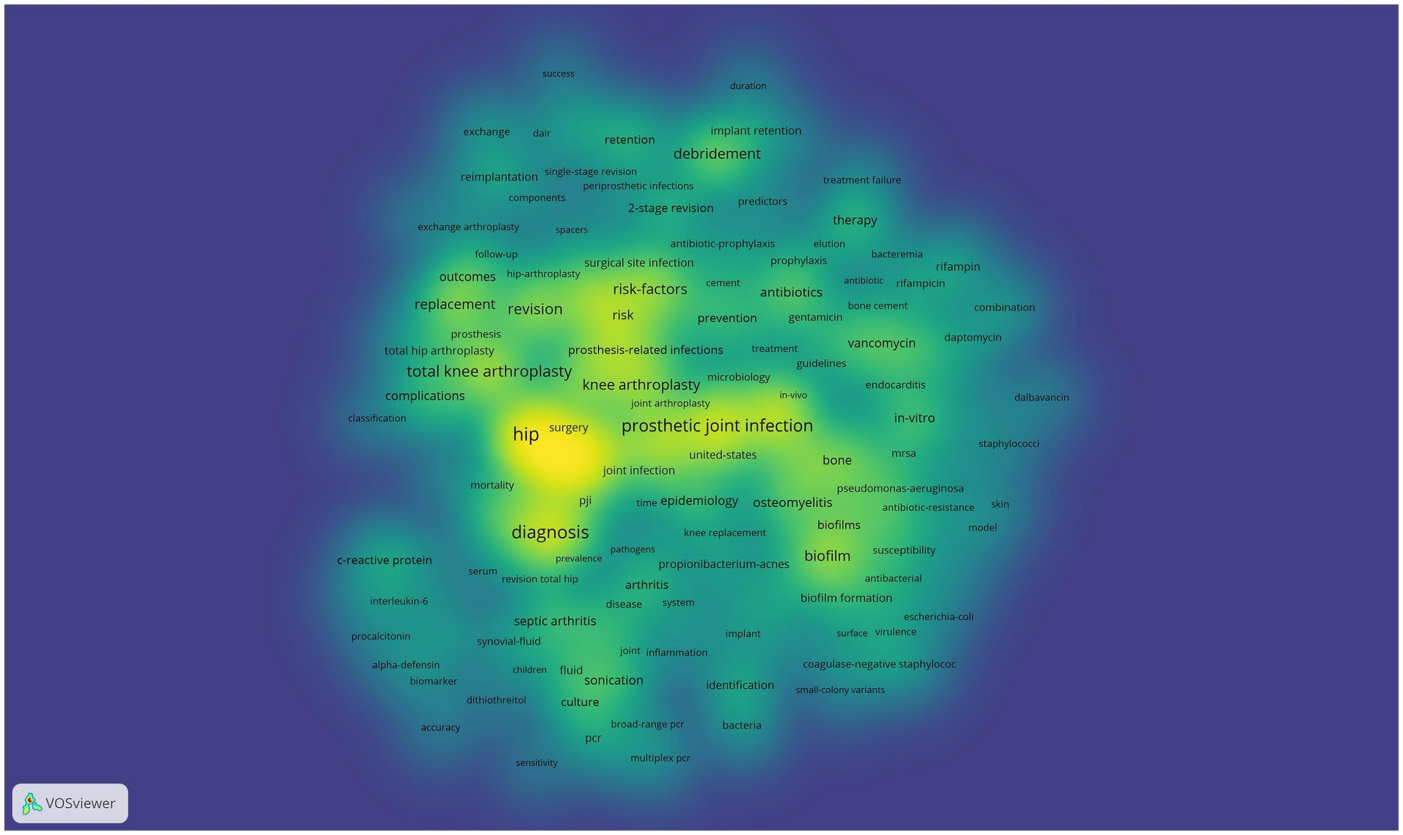
Mapping of countries of studies related to PJI.

Keywords are commonly used in publications to summarize research topics, and their analysis can reveal the hotspots and directions of research in a particular area. Keywords related to prosthetic joint infections (*n* ≥ 70) are shown in Table 5. Among these keywords, periprosthetic joint infections occurred most frequently (*n* = 314), followed by prosthetic joint infections (*n* = 311), hip (*n* = 301), infection (*n* = 288), diagnosis (*n* = 241), and arthroplasty (*n* = 232). The centrality of joint infection (*n* = 44, centrality = 0.08), resurfacing (*n* = 48, centrality = 0.07), cementing (*n* = 47, centrality = 0.06), and C-reactive protein (*n* = 54, centrality = 0.06) was greater than 0.05, suggesting that these keywords have greater than 0.05 centrality in the artificial joint infection field of importance. Figure 5 shows the high-frequency keywords on the density map. The intensity of the colors is proportional to the frequency of the keywords in the publications.

**Table 5:** Keywords related to PJ (*n* > 70).

Count	Centrality	Year	Keywords
314	0.01	2014	Periprosthetic joint infection
311	0.01	2013	Prosthetic joint infections
301	0.01	2013	Hip
288	0.02	2013	Prosthetic joint infection
241	0.01	2013	Diagnosis
232	0.01	2013	Arthroplasty
217	0.02	2013	Total knee arthroplasty
192	0.01	2013	Total hip arthroplasty
172	0.02	2013	Management
164	0.03	2013	Risk factors
162	0.03	2013	Knee arthroplasty
157	0.03	2013	*Staphylococcus aureus*
147	0.02	2013	Revision
129	0.01	2013	Debridement
122	0.01	2013	Replacement
121	0.03	2013	Total hip
108	0.03	2013	Risk
90	0.04	2013	Septic arthritis
88	0.03	2013	Bone
87	0.03	2013	*in vitro*
86	0.03	2014	Prosthetic joint infections
78	0.02	2015	Infection
77	0.03	2013	Therapy
75	0.00	2013	Retention
75	0.02	2013	Efficacy
74	0.04	2013	Prosthesis-relatedinfections
70	0.03	2013	Vancomycin

Keywords with strong citation bursts are those that are frequently cited over a given period of time. Thus keyword citation bursts can help track the rise and fall of research hotspots. The blue line indicates the time interval and the red line indicates the time from start to finish of the keyword. As shown keywords with citation bursts first appeared in 2013 with “treatment failure” having a strong burst (strength = 4.9) followed by “septic arthritis” (strength = 4.7) and “bacteria” (strength = 4.53). The most recent keywords to be cited in the outbreak were “period” and “system” which appeared in 2021. Changes in the keyword citation explosion reflect changes in research trends.

**Figure 6:**
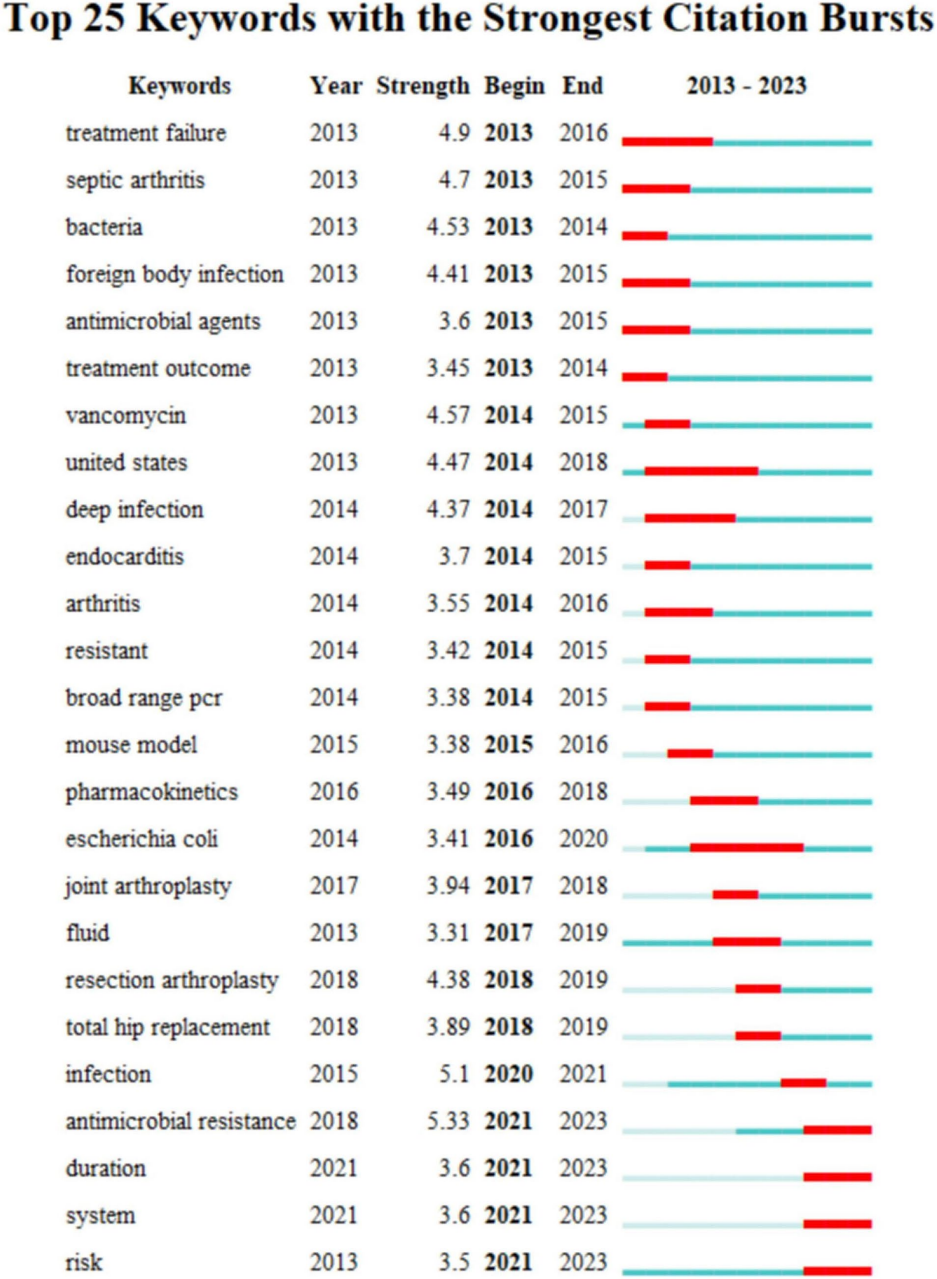
High-frequency keywords for PJl.

The timeline mapping of keywords is used to display the high frequency keywords in each cluster. As shown in Figure 6 the color bar in the lower left corner indicates the time range in which the keywords appear with the colors transitioning from dark blue (2013) to red (2023). The color of the keywords indicates how often they appear in the corresponding year. Keywords regarding prosthetic joint infections were grouped into the following eight clusters: #0 debridement #1 staphylococcus epidermidis #2 gentamicin #3 prosthesis-related infections #4 periprosthesis-related infections #5 procalcitonin #6 knee arthroplasty and #7 septic arthritis. In 2015 research focused on surgery-related infections. In particular the mechanisms and treatment of prosthesis-related infections and bacterial infections (e.g., staphylococcus). Keywords such as “knee arthroplasty” and “debridement” showed greater importance in 2013–2015 implying that at that time the prevention and management of infections after joint replacement were key topics. Infection prevention and management were key topics at that time. The yellow and orange areas of the graph reflect the research hotspots from 2016 to 2020. At this time new research directions such as “gentamicin” “antimicrobial resistance” “biomarker” and “antibiomarker” are emerging. Biomarker and other topics began to appear gradually, indicating that the focus of research gradually expanded from the surgery itself to antimicrobial therapy and drug resistance issues. As we move through the red and crimson areas we can see that research in recent years has focused on more specific areas such as “periprosthetic joint infection” and “septic arthritis.” septic arthritis.” These keywords indicate a gradual progression of research into more refined diagnostic and therapeutic approaches to the mechanisms of inflammation following joint infection. At the same time the increased use of biomarkers and molecular diagnostic tools such as “biomarker” in recent studies reflects the field’s continued efforts to address difficult infections.

### 3.8 Analysis of article global citations (GCS)

**Figure 7:**
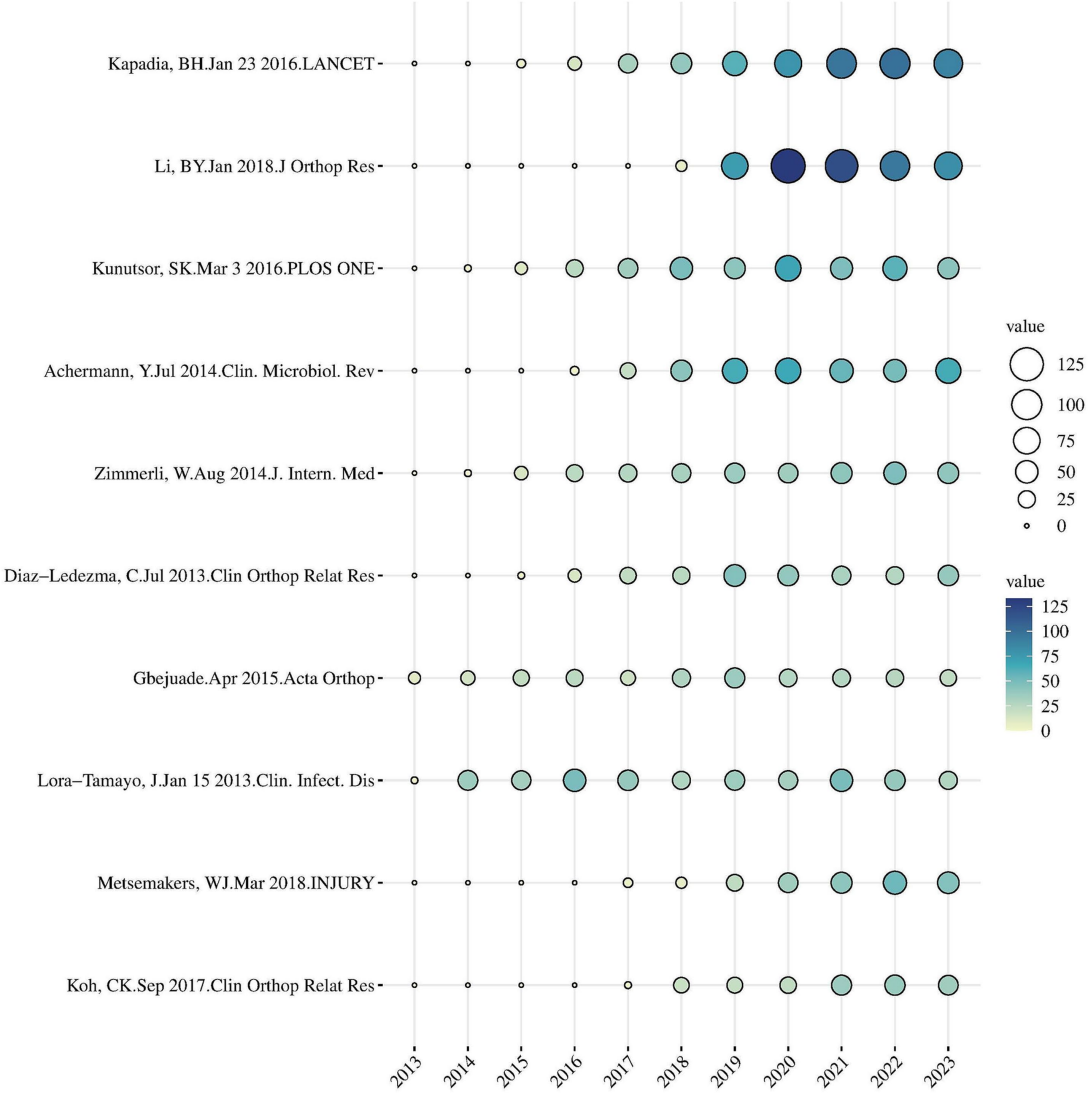
Yearly number of global citations of articles with high global citations (GCS), the GCS of each article is shown by the size and color of the circle.

In Figure 7, the yearly GCS data for the top 10 articles are displayed. The most often referenced paper, “Periprosthetic Joint Infection” authored by Andrea B. Nelson et al. and published on July 12, 2023, asserts that bacterial infections, such as *Staphylococcus aureus*, are the main causal agents of periprosthetic joint infections (PJIs). Staphlococcus aureus. One of the processes implicated is the development of a biofilm by the bacteria on the surface of the prosthesis, therefore impeding the infection clearance process. Therapeutic approaches are categorized into conservative antibacterial therapy and surgical intervention, with the latter involving debridement and prosthesis replacement. Prevention measures encompass preoperative optimization, asepsis during surgery, and antibiotic prophylaxis. The present study is investigating innovative antimicrobial-coated prostheses and topical antimicrobial treatments in order to reduce the development of biofilm on prosthetic surfaces (Nelson et al., 2023).

In “Bacteria antibiotic resistance: New challenges and opportunities for implant-associated orthopedic infections,” Li Bingyun et al. discuss the threat of antibiotic-resistant bacteria, particularly MRSA, in orthopedic surgeries. These infections increase patient mortality and treatment costs. The article highlights the issue of biofilm formation on implants, which reduces the effectiveness of traditional antibiotics. The authors stress the need for innovative non-antibiotic approaches, such as new materials and technologies, to prevent bacterial adhesion and growth on medical implants (Li and Webster, 2018).

In “*Propionibacterium acnes*: from Commensal to Opportunistic Biofilm-Associated Implant Pathogen,” Kunutsor, Setor K. et al. explore the behavior of *Propionibacterium acnes* (now known as *Cutibacterium acnes*), a typically benign skin commensal that can become an opportunistic pathogen in infections associated with implants. Infections caused by *Propionibacterium acnes* occur when it develops biofilms on medical devices, including shoulder prosthesis, cerebrovascular shunts, and cardiovascular implants. With the advancement of molecular detection methods such as ultrasound cleaning and 16S rRNA gene PCR, the ability to detect these infections has been enhanced. Although *Propionibacterium acnes* is highly sensitive to a variety of antibiotics (such as beta-lactams, quinolones, and rifampin), its resistance to clindamycin is gradually increasing. Effective treatment often includes surgical removal of the infected implant and a long-term antibiotic therapy lasting 3 to 6 months. Rifampicin has shown potential in treating *Propionibacterium acnes* biofilms, but further research is needed to confirm its effectiveness in combination therapy (Kunutsor et al., 2016).

In “Patient-Related Risk Factors for Periprosthetic Joint Infection after Total Joint Arthroplasty: A Systematic Review and Meta-Analysis,” Achermann, Y et al. conducted a comprehensive review and analysis of risk variables associated to patients who are at risk of developing periprosthetic joint infection (PJI) following total joint arthroplasty (TJA). The study identified the following key elements as substantial contributors to the higher risk of PJI: ① Obesity (excessive BMI). ② Diabetes (particularly suboptimal management). ③ Tobacco use: Smokers face an increased susceptibility to infection as a result of compromised tissue regeneration and immunological mechanism. ④ Aging: Older individuals are more susceptible to PJI, partly because of a compromised immune system and other concurrent medical conditions. ⑤ Immunosuppression: Patients undergoing immunosuppressive treatment, such as those with autoimmune diseases, are more prone to developing PJI compared to those who are not. Immunosuppressive therapy, such as in the treatment of autoimmune disorders or following organ transplantation, increases the susceptibility of patients to infection due to compromised immunological resistance. ⑥ Insufficient nourishment. Furthermore, the presence of comorbidities such as a previous joint surgery, renal disease, and chronic liver disease is linked to a higher susceptibility to infection. The article underscores the need of recognizing and controlling these patient-related variables in the clinic to avoid surgical complications and enhance patient prognosis (Achermann et al., 2014).

An paper by Zimmerli, W et al. titled “Clinical presentation and treatment of orthopaedic implant-associated infection” specifically addresses the symptoms, diagnosis, and management of infections linked with orthopaedic implants. The article specifically addresses the symptoms, diagnostic methods, and therapeutic approaches relevant to orthopedic implant-associated infection. Infections of this nature provide a substantial challenge in the field of orthopedic surgery and have the potential to result in implant failure, extended healing time, and the necessity for supplementary procedures. The paper highlights that infections related to orthopedic implants are intricate and necessitate coordination among many disciplines, including timely diagnosis, suitable antibiotic treatment, and surgical intervention. Effective prevention measures and timely intervention are crucial for enhancing patient prognosis and minimizing the likelihood of implant failure. The significance of timely diagnosis and thorough therapy is underscored (Zimmerli, 2014).

The 10 investigations on prosthetic joint infections primarily examined two prominent trends: firstly, the major areas of investigation were antibiotic resistance and biofilm development. Furthermore, in the clinical context, research have highlighted the significance of identifying, preventing, and adopting a multidisciplinary approach to treatment. Lora-Tamayo J conducted a study on the efficacy of treating infections in prosthetic joints, underscoring the present necessity for specialists from many disciplines to collaborate in managing complex infections, particularly in challenging situations (Diaz-Ledezma et al., 2013).

The number of detailed investigations of particular bacteria, such *Propionibacterium acnes*, is also growing. Analysis of over 11,000 knee arthroplasties conducted by Koh CK revealed that infection is a significant factor contributing to surgical failure (Koh et al., 2017).

These trends show the multidimensional development of research from basic microbiology to clinical applications.

### 3.9 Co-cited reference analysis

**Figure 8:**
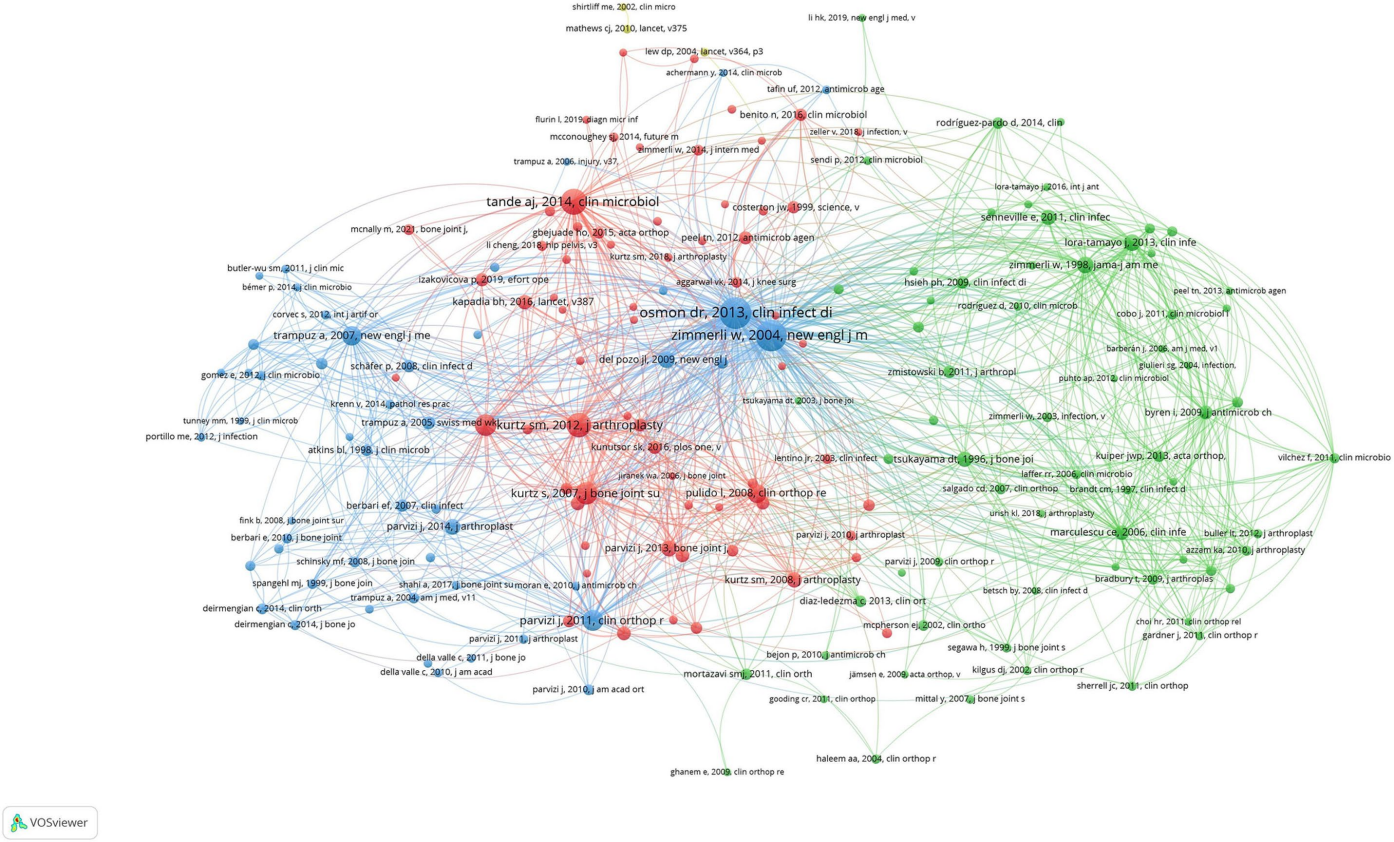
Reference network of PJI-related studies with common citations.

A co-cited paper is two papers that are cited by another identical paper, and co-citation analysis can be used to track the evolution of a particular field. Co-citation networks focus on identifying research topics that are closely related to a specific discipline, distinguishing them from the broader approach of global citation analysis (Castañeda-Reyes et al., 2020). Due to the high citation frequency, the minimum number of citations per article was set at 26. The literature search yielded a total of 41,000 articles, of which 185 were selected for co-citation analysis, as shown in Figure 8. The line connecting two nodes indicates that these two nodes were co-cited in the same article, and the shorter the line indicates a closer relationship. The size of the node represents the total link strength and the number of co-citations for that article. In addition, different colors are used to classify articles into different clusters. The cited literature was divided into clusters, each focusing on a particular aspect of prosthetic joint infection.

This co-citation network demonstrates the different research directions and subfields in the study of artificial joint infections. The red cluster of literature focuses more on clinical manipulation and treatment, the green cluster focuses on pathogens and mechanisms of infection, and the blue literature focuses on diagnostic and therapeutic strategies for infections. The red node (Cluster 1) contains 67 documents that focus on infection research related to artificial joint replacement. Larger nodes such as Kurtz SM, 2012 and Parvizi J, 2011 represent important studies that are frequently cited in the field of artificial joint replacement and infection management. The green node (Cluster 2) contains 66 publications, and the majority of the literature in this cluster deals with clinical infection control and microbiology research, especially the role of bacterial infections in artificial joint implantation. Literature such as Zimmerli W, 2004 and Lora-Tamayo J, 2013 are more prominent in this area. The blue node (Cluster 3) contains 49 papers that focus on the prevention, diagnosis, and treatment of infections, especially for chronic infections of bone and joints. This figure reflects the multidisciplinary nature of the field.

## 4 Discussion

This study reveals the major trends and hotspots in global research on prosthetic joint infection (PJI) between 2013 and 2023 through bibliometric analysis. Through the bibliometric analysis of the literature, we found that despite the remarkable progress in PJI research over the past decade, there are still many challenges and unanswered questions regarding diagnosis, treatment, and prevention. The following aspects deserve further discussion.

### 4.1 Global trends in research on prosthetic joint infections

First, this study confirms a significant growing trend in the field of prosthetic joint infection research, especially peaking in 2021. This phenomenon reflects the complexity of PJI in the clinical setting and its impact on patient quality of life. The United States is particularly dominant in PJI research, and it has frequent international collaborations with other countries such as Germany and Switzerland. In contrast, although the number of studies in this area has increased in China and Canada, the international impact of the research is relatively low. This suggests the need for more international multicenter collaborations in the future to promote the widespread application of research results and academic impact.

### 4.2 Changes in research priorities

Keyword co-occurrence analysis showed that the study of biofilm-associated infections, antibiotic resistance, and novel biomarkers became the core hotspots in the field of PJI. In recent years, the mechanism of biofilm formation has been regarded as one of the main reasons why PJI is difficult to cure (Juodeikis and Carding, 2022). This is in line with several high-impact studies in recent years, which have shown that the presence of biofilms greatly reduces the efficacy of antibiotics, especially in infections with multiresistant bacteria (e.g., MRSA). Future research should focus on how to effectively disrupt biofilm structures as well as develop new topical antimicrobial therapies to reduce the impact of antibiotic resistance (Donlan and Costerton, 2002).

Our study highlights that enhancing diagnostic methods maybe become a significant breakthrough area in the coming years. Although the current diagnosis of PJI relies on conventional serologic tests and bacterial cultures these methods lack adequate sensitivity and specificity (Kim and Cho, 2021). Recent research on novel biomarkers for PJI detection has highlighted several emerging trends and focal points: ① Cytokines and chemokines: Cytokines such as IL-6 IL-10 and TNF-*α* and chemokines such as the CXC-gene family (Liu et al., 2021); ② Metabolite markers: Lipid metabolites amino acids and their derivatives (Mecatti et al., 2020); ③ Exosomal miRNA: Exosomal miRNAs including miR-223 and miR-146a which regulate immune and inflammatory responses with miR-223 suppressing dendritic cell maturation and miR-146a modulating NF-κB pathway inflammation (Asgarpour et al., 2020); ④ α-defensins: Recent research demonstrates that α-defensins respond to a wide range of microbial infections including Gram-positive and Gram-negative bacteria as well as fungi. In cases of PJI alpha-defensin levels in joint fluid are significantly elevated providing a reliable biomarker for infection presence. Studies indicate that alpha-defensin testing achieves a sensitivity of 94.4% and a specificity of 89.5% for PJI diagnosis which underscores its clinical utility as a rapid and accurate diagnostic tool (Deirmengian et al., 2015). These biomarkers may provide potential methods for faster more accurate PJI diagnosis. Further clinical studies are needed to verify their diagnostic value across different infection stages (Zilberman and Elsner, 2008).

Furthermore, co-citation analysis has shown that while there is a substantial body of research on the early detection and treatment of periprosthetic infections, there remains a dearth of comprehensive attention on the long-term prevention and longitudinal monitoring of infections (Schwarz et al., 2019). The MSKI consensus guideline, published by the Musculoskeletal Infection Society, is an internationally recognized standard for the diagnosis and management of prosthetic joint infections (PJI). It provides a structured framework for PJI diagnosis by combining clinical symptoms, serum biomarkers (e.g., CRP and ESR), and microbiological culture results to enhance diagnostic accuracy. The guideline also offers evidence-based recommendations for treatment, emphasizing staged surgical interventions and specific antibiotic regimens tailored to infection severity and pathogen type. Internationally, the MSKI guideline has been widely adopted, significantly contributing to standardized care practices and improving clinical outcomes in PJI management across different healthcare systems.

Undoubtedly, future research should delve deeper into personalized therapy alternatives, particularly targeted treatment approaches for high-risk patient populations, in order to enhance the long-term outlook of patients with PJI.

### 4.3 Global trends in prevention, diagnosis, and treatment

In terms of prevention: ① Surgical implants coated with antimicrobial agents have shown considerable promise in reducing the incidence of postoperative infections (Onorato et al., 2024). A key area for future research will be to integrate cutting-edge material science and technology to further study the long-term impact of antimicrobial coatings and their broad effectiveness against various bacterial strains (Romanò et al., 2015). In North America and Europe, research and application of such antimicrobial-coated implants have been increasing annually (Akay and Yaghmur, 2024). ② Sterile techniques in the operating room have made significant advancements from 2013 to 2023, including air purification and ultraviolet germicidal methods. The use of pulsed xenon ultraviolet lamps for environmental disinfection in operating rooms has been proven to reduce the presence of bacteria and viruses within the surgical environment, thereby decreasing the rate of hospital-acquired infections (Simmons et al., 2017). Studies have shown that UV-C radiation has a potent germicidal effect on a variety of microorganisms, including viruses, methicillin-resistant *Staphylococcus aureus* (MRSA), and vancomycin-resistant enterococci (VRE). UV-C achieves sterilization by disrupting the DNA of microorganisms, thereby reducing the rate of hospital-acquired infections and contamination (Ramos et al., 2020).

In terms of diagnostics, molecular diagnostic technology has become a research hotspot in the diagnosis of periprosthetic joint infections (PJI). Novel molecular markers, such as *α*-defensins and exosomal miRNA, have shown high specificity and sensitivity in the early diagnosis of PJI. Studies have shown that the detection of α-defensins exhibits strong diagnostic capabilities for a range of microbial infections (de Oliveira Dias and Franco, 2015). Concurrently, exosomal miRNAs, such as miR-223 and miR-146a, offer novel insights into the early diagnosis of periprosthetic joint infections (PJI) by modulating immune responses (Mortazavi-Jahromi et al., 2020). Additionally, molecular diagnostic tools like 16S rRNA sequencing have demonstrated significant advantages in rapid pathogen detection, especially when traditional methods are limited (Johnson et al., 2019). With the advancement of molecular techniques, future research can further assess their broad application value in PJI diagnosis. Several countries in Europe, including Germany and the Netherlands, have begun to promote the use of PET-CT for the diagnosis of Periprosthetic Joint Infection (PJI). By labeling specific metabolites, such as FDG, it can display strong signals at the sites of infection, making it particularly suitable for the diagnosis of chronic low-grade infections (Hu et al., 2022). Research teams in some countries, such as the United Kingdom, are exploring ultrasound enhancement techniques, using microbubble contrast agents to improve the accuracy of ultrasound in the early diagnosis of infections, which is suitable for early or low-grade infections (Henriquez-Camacho et al., 2015).

In terms of treatment, the formation of biofilms is a challenge in the management of PJI. Various biofilm inhibitors and disruptors, such as silver ions and sodium citrate, are under development with the aim of breaking down biofilms to enhance the efficacy of antibiotics. In clinical trials in the United States and Italy, sodium citrate and some novel enzymatic agents have shown good biofilm disruption effects, significantly enhancing the permeability of antibiotics, thereby effectively reducing the infection rate of drug-resistant bacteria (Taha et al., 2018). Immunomodulatory therapy has begun to be applied in the treatment of PJI globally, by modulating the patient’s immune response to enhance the ability to control infections. Researchers in Japan and the United States have explored the use of cytokine modulators, such as IL-6 inhibitors, to suppress inflammatory responses and reduce the damage of chronic inflammation to prosthetic joints (Ohsugi, 2019). With the increase in infections caused by drug-resistant bacteria such as MRSA and VRE, the effectiveness of single-antibiotic regimens is diminishing, making combination antibiotic therapies a hot topic of research. Research teams in the United States are investigating the combined use of multiple antibiotics, such as vancomycin and daptomycin, to enhance therapeutic outcomes (Rose et al., 2021). A multicenter study in Europe involving over 600 PJI patients demonstrated that combination antibiotic therapy significantly reduced the recurrence rate of drug-resistant bacterial infections, particularly in patients with MRSA infections. Furthermore, the study found that combination therapy could reduce the dosage of antibiotics and decrease side effects (Roger et al., 2024).

### 4.4 Limitations and prospects of the study

The bibliometric analysis of this study was conducted through the Web of Science Core Collection database, and although it provided studies of PJI worldwide, limitations of the data sources (e.g., literature in all languages or databases were not covered) may have affected the comprehensiveness of the results. In addition, although bibliometric analysis can reveal research hotspots and trends, it cannot delve into the specific mechanisms and clinical implications behind various types of studies.

## 5 Conclusion

Globally, countries are exhibiting a diversified trend in PJI diagnostic technologies, ranging from molecular diagnostics to enhanced imaging, novel biomarkers, and AI-assisted systems. These innovative methods significantly improve diagnostic accuracy and efficiency, providing new avenues for earlier and more precise PJI diagnosis. Similarly, the treatment of prosthetic joint infections is moving toward integrated and personalized approaches. Emerging treatment strategies such as biofilm disruptors, local antibiotic delivery systems, and multidisciplinary collaboration demonstrate promising prospects. Additionally, the rise in antibiotic-resistant bacteria is driving researchers to explore combined antibiotic therapies and immunomodulatory treatments. In terms of prevention, diagnostic standards vary across countries, while advancements in antimicrobial-coated prostheses and novel sterile operating room technologies have proven effective in reducing postoperative infection rates.

## Data Availability

The original contributions presented in the study are included in the article/supplementary material, further inquiries can be directed to the corresponding author.
